# Development of Ferrero Nutrition Criteria: category-specific, progressive guidelines for product innovation

**DOI:** 10.3389/fnut.2025.1612183

**Published:** 2025-07-14

**Authors:** Federico Canzoneri, Mélanie Charron, Adam Drewnowski, Emanuela Alzari, Marta Biolatti, Emanuela Cavalli, Marco Franci, Stefania Giri, Fritz Maingrette, Maria Malgorzata Maj, Ileana Manera, Ginevra Rosso, Vittoria Russo, Gloria Veneziani, Sebastiano Collino, Enrico Pavesi

**Affiliations:** ^1^Ferrero Group, Soremartec Italia Srl, Alba, Italy; ^2^Center for Public Health Nutrition, University of Washington, Seattle, WA, United States

**Keywords:** nutrient profiling, nutrition criteria, innovation, product formulation, nutrient density, energy density, nutritional quality

## Abstract

**Background:**

Nutrition standards developed for internal use can help assess nutrient density and guide improvements in company product portfolios. Often category-specific, such standards are used to guide product innovation and new product development.

**Objective:**

To develop Ferrero Nutrition Criteria (FNC), a new set of progressive nutrition standards for internal use to evaluate product portfolio and to guide product formulation and development.

**Methods:**

The FNC was developed with reference to a publicly available database, the Food and Nutrient Database for Dietary Studies (FNDDS 2017-18) and the corresponding Food Pattern Equivalents Database (FPED), both maintained by the US Department of Agriculture. Nutrition standards were based on recommendations issued by international agencies and other expert bodies, pledges that regulate advertising and marketing to children, other existing nutrient profiling models, consumer trends, technological constraints, the nutritional composition of Ferrero products and their taste performance. Minimum values for nutrients and ingredients to encourage were based on dietary guidelines.

**Results:**

The FNC model was used to assign foods and beverages into three classes that were largely based on energy and on nutrients to limit (added sugar, saturated fat and sodium). Among nutrients to encourage were protein, fiber, vitamins, minerals and among ingredients to encourage were dairy, nuts, fruits, whole grains, legumes, seeds and vegetables. The setting of category specific criteria also took into account taste, portion size, eating occasions, and role of the food or beverage in the overall diet. The FNC model is intended to be progressive and to evolve continually as new products are developed.

**Conclusion:**

The FNC model reflects Ferrero’s commitment to transparency and the promotion of responsible consumption. It will be used to guide improvements in the Ferrero product portfolio.

## 1 Introduction

The four core principles of a healthy diet are adequacy, moderation, diversity, and balance. The recent joint statement by the Food and Agriculture Organization of the United Nations (FAO) and the World Health Organization (WHO) (1) defined dietary adequacy as consuming enough essential nutrients to prevent nutrient deficiencies and to promote optimal health. Moderation meant limiting the consumption of foods, nutrients, or components associated with adverse health outcomes. Balance referred to optimal proportions of macronutrients, whereas dietary diversity was the consumption of a wide variety of nutrient-rich foods both across and within food groups (1). These core principles apply to healthy dietary patterns and not necessarily to individual foods (1).

The joint FAO/WHO statement (1) recognized that dietary patterns are subject to multiple influences. Among the social, economic, and environmental drivers of food choice are taste, cost, convenience, health, variety, and growing concerns with the environment (2). It emphasized that diets need to be adequate in both energy and nutrients (1). Also highlighted as the importance of dietary moderation and the need to limit saturated fat, free sugars, and sodium. Aligning with previous WHO positions, the stated goal was to restrict both saturated fats and free sugars to less than 10% of daily energy (3, 4) and to limit dietary sodium to less than 2 g/day, equivalent to < 5 g/day of salt (5).

Dietary diversity and balance, both within and across food groups, were recognized as additional measures of diet quality (6). Dietary diversity was viewed as a means to ensure sufficient consumption of a broad range of micronutrients, trace elements, and bioactive compounds (1). For example, the Minimum Dietary Diversity Score for Women (M-DDW), cited in the joint FAO/WHO statement, gives positive scores to dairy foods, fruit, grains and legumes when consumed over the previous 24 h (7, 8). Those are the typical nutrient-dense food groups to encourage.

It is often said that there are no bad foods, only bad diets (9, 10). The American Dietetic Association position (11) that “all foods can fit” is and remains a core principle of dietetic practice. The 2010 Dietary Guidelines for Americans (DGA) launched the concept of “discretionary calories” (10), defined as the daily calories remaining for consumption after all the essential nutrient needs have been met. These “extra” calories could then be used for enjoyment and indulgence (10). The DGA recommended that these calories should not exceed 10–15% of the total energy intake (10); however, that amount could be increased by augmenting physical activity and eating more nutrient-rich foods (6). For example, a diet optimization study showed that indulgent foods could account for up to 20% of market basket weight (40% of energy), provided that the diet also contained about two thirds of the most nutrient-rich foods by weight (12). Energy-dense foods and nutrient-dense foods can both fit in a healthy, balanced, diverse, and nutrient adequate diet (1).

Different methods are used to assess diet quality and to track nutrient density of individual foods. Nutrient profiling models (NPMs) do not capture total diet quality but can be used to assess nutrient content of individual foods per reference amount. Initially intended for use by supermarkets and for front-of-pack labels (13), their development was motivated by the need to convey information about the relative healthfulness of foods to the consumer (13). Since then, NPMs have become the basis for assessing innovation and product formulation by the food industry. Several companies have developed internal NPM to screen product portfolios and to assist in the development of new foods and beverages (14–19). Furthermore, the role and degree of processing has also been used to classify foods (20–26). While NPMs differ from the level of processing and their approaches are also different, the topic continues to be debated within the scientific community (27). The variation of nutritional value and nutrient-dense foods across different levels of processing is still under investigation (28, 29). The recent report by IUFoST provides a detailed status of this complex research area (30), where further attempts at sub-categorization may take place in the future.

Innovations in the category of packaged snacks, the focus of this report, are particularly challenging. Snacks need to be tasty, pleasurable, and convenient to use, they can also contribute to a healthy diet by providing desirable nutrients and ingredients of interest. Currently, all snacks, including packaged snacks, provide about 20% of daily energy in the US diet (31) and an estimated 150–200 kcal per serving. In European countries, between-meal snacks contribute to approximately 24% of the daily energy intake in younger children, with each serving providing an estimated 180–220 kcal (32).

Ferrero has developed its own set of standards, introduced here as the Ferrero Nutrition Criteria (FNC). The FNC will serve to guide and monitor nutritional goals and commitments that are aligned with Ferrero’s portfolio and its sustainability agenda. The proposed FNC will evolve with time and will provide a foundation for continuing product innovation and development. This paper provides an in-depth description of the criteria used in FNC development.

## 2 Materials and methods

### 2.1 Development of Ferrero Nutrition Criteria

The present goal was to develop a category-specific, progressive NPM to help guide the formulation of new foods and beverages and to track the nutritional composition of the product portfolio. The development of FNC involved extensive research and consultation with nutrition experts and food science specialists with direct knowledge of technical feasibility and product formulation and development. A cross-functional team that included internal nutrition scientists, food technologists as well as external independent academic nutrition scientist advisors was convened.

The concept of nutrient density was fundamental to the development of the FNC. Nutrient-rich foods are those that contain more nutrients than calories; foods that are nutrient poor contain more calories than nutrients (33). Nutrient density of foods can be improved by reducing their content of saturated fat, free sugars, or sodium, as suggested by the WHO (1). Nutrient density can also be improved by adding desirable nutrients, ingredients, or bioactive compounds to new or reformulated products. The WHO has promoted the inclusion of milk and dairy, nuts, fruits, whole grains, legumes, and vegetables in new or reformulated products to improve their overall nutritional value (34). Similarly, the US Food and Drug Administration (FDA) has proposed that to be considered “healthy,” foods would need to contain minimum quantities of whole grains, dairy, fruits and vegetables, while strictly limiting added sugars, sodium, and saturated fats. The proposed criteria for “healthy” foods were calculated per serving size, defined by the FDA as the reference amounts customarily consumed (RACC) (35). Many of these criteria were adopted by the FNC.

The new FNC was developed with reference to a nutrient composition database, maintained by the US Department of Agriculture (USDA) and publicly available on FoodData Central (36). The Ferrero food categories of interest used for the development of the FNC were: edible-ices, fine bakery wares, chilled products, beverages, sugar confectionery, confectionery, protein bar, fruit and nut snacks. Those categories are also included in the FNDDS 2017-18. The first analytical goal was to assess energy and nutrient content of foods by category and to provide estimates of portion sizes. Ferrero products were then compared to foods available in the US marketplace. Subsequently, the new FNC were developed taking into consideration many critical factors, including global dietary guidelines (3–5, 37), pledges that regulate advertising and marketing to children (38–41), other existing NPMs (14–17, 42, 43), consumer trends, technological constraints, the nutritional composition of Ferrero products and their taste performance.

### 2.2 The food and nutrient database for dietary studies FNDDS 2017-18

The What We Eat in America (WWEIA) study is the dietary component of the National Health and Nutrition Examination Survey (NHANES). The present analysis of energy content and nutrient composition of foods came from the USDA Food and Nutrient Database for Dietary Studies (FNDDS 2017-18) (44), which contains foods consumed by NHANES participants. Individual food items in the FNDDS 2017-18 (identified by 8-digit codes) are aggregated by the USDA into food groups, food categories, and food subcategories using WWEIA 1-digit, 2-digit and 4-digit codes (45).

The 4-digit codes for food subcategories were provided by the WWEIA (45) and were the same as used in the Dietary Guidelines for Americans (DGA). The categories were edible ices (ice cream, frozen desserts, gelatins, ices, sorbets); bakery goods (doughnuts, sweet rolls and pastries, cakes and pies, cookies and brownies); chilled products (pudding); beverages (non-dairy drinks); sugar confectionery (candy without chocolate); confectionery (nut spread and candy with chocolate); protein bars (nutrition bars); fruit and nut snacks (dried fruits, nuts and seeds). The FNDDS 2017-17 was then matched with the corresponding Food Patterns Equivalents Database (FPED) 2017-2018, which converts FNDDS foods into 37 USDA Food Patterns components. These are expressed as cup equivalents of fruit, vegetables, and dairy, ounce equivalents of grains, and teaspoon equivalents of added sugars. The FPED was formerly known as the MyPyramid Equivalents Database. The FNDDS database is for the most part unbranded and does not include an electronic ingredient list.

### 2.3 Ferrero category-specific approach

Across-the-board NPMs apply the same nutrient standards to all foods. By contrast, category-specific models assess the relative nutrient density of foods within a given food group, subgroup, or product line. Early NPMs, including the FSA-Ofcom model (later Nutri-Score) (46, 47), the Health Star Rating (HSR) (modified FSA-Ofcom model) (46, 48), and the Nutrient Rich Food (NRF) index (49) are across-the-board models. Both models identify vegetables and fruit as the most nutrient-rich food groups, but some fail to make finer discrimination within groups.

A category-specific approach recognizes that not all foods contain the same array of nutrients. For instance, fruits generally do not contain calcium, whereas dairy does not contain vitamin C. As a result, the nutritional criteria for a snack product would not be the same as those for milk or dairy, given inherent differences in ingredients, energy density and consumption patterns. Different categories of foods, such as beverages, snacks, and confectionery, vary significantly in their nutritional composition, consumer expectations, and contribution to the total diet. Category-specific NPMs are more closely aligned with consumer needs, helping to identify the most nutrient-rich options within a given category.

Category-specific NPMs can better support innovation in the food industry where there is a need to balance desired nutritional quality with consumer preferences. A category specific model allows product developers to innovate and reformulate within a structured framework. The setting of progressive nutrition targets by product class enables food manufacturers to improve nutrient quality of their product lines without compromising on taste, cost, or convenience. This targeted approach spurs the development of healthier options within each food category. Each food type is evaluated against relevant and appropriate standards.

Specifically, the Ferrero categories are: edible ices (ice cream milk-based, water ices and sorbets), fine bakery wares (ambient bakery, sweet biscuits), chilled (chilled snacks), beverages (ready-to-drink tea), sugar confectionery (hard candy, soft candy), confectionery (spreads, chocolate confectionery, praline and bites), protein bar (low-sugar protein bar), fruit and nut snacks (fruit and nut bars and trail mix). It is important to distinguish between chilled snacks and edible ices to better understand their storage implications. Chilled snacks are food items that need to be stored at low temperatures, typically above freezing but below room temperature, to maintain their freshness and safety. These products are typically found in the refrigerated section. In contrast, edible-ices include frozen items like milk-based ice creams and water ices that require storage below freezing to preserve their texture and safety. These product categories were matched to the corresponding food groups and subgroups in the FNDDS 2017-18 database.

### 2.4 Base of calculation per caloric target

The nutrient density of foods is typically calculated based on a reference amount, which can be 100 g, 100 kcal, or serving size (13). Selecting the basis for calculation is often influenced by local regulatory requirements. For instance, in the United States, food regulations and labels are based on serving sizes defined as RACC (50). In contrast, dietary information in the European Union is provided per 100 g. However, the 100 g reference amount is not suitable for pre-packaged individual snacks, which generally weigh much less. The serving size for snacks plays a key role, as they are often individually packaged for each eating occasion, promoting responsible consumption. It is generally accepted that the snacks should contain between 150 and 250 kcal, preferably under 200 kcal. Throughout 2022/2023, the vast majority of Ferrero products (97.1% by marketed volume) contained 200 kcal or less per serving, with a significant proportion (91.4%) containing 150 kcal or less per serving (51). The decision to base the FNC on a caloric target per serving ensures that its criteria are both challenging and achievable. In addition, this approach provides flexibility in the development process and formulations, while also considering a caloric limit specific to each product category and tailored to its likely consumption.

### 2.5 Selection of age-appropriate daily reference value

Public health nutritional guidelines specific to different age groups were used to determine daily values (DV). As part of this process, children aged 3–8 years were identified as a distinct group with lower daily energy needs and special nutritional requirements. The WHO most recent recommendations for total fat, saturated fat, trans-fat, added sugar, protein, dietary fiber, and sodium (3–5, 37, 52, 53) were used to establish the FNC daily values for an energy intake of 2,000 kcal/day for adults. The same WHO recommendations were used to establish nutrient standards for children aged 3–8 years but were scaled down to 1,300 kcal/day (3–5, 52, 53). For children, dietary fiber standards follow those recommended by the European Food Safety Authority (EFSA) (54). For both target populations, the nutrient reference values (NRV) for micronutrients were based on European Regulation (55). The DV and NRV for nutrients to limit and to encourage are summarized in Table 1.

**TABLE 1:** Daily values (DV) and nutrient reference values (NRV) of various nutrients to limit and nutrients to encourage, for adults and children (9 + years), and children aged 3–8 years.

Nutrient	Unit	Adults and children (9 + years)	Children (3–8 years)
**Nutrients to limit**		**DV**	**DV**
Energy	kcal	2,000	1,300
Total fat	g	67	43
Saturated fat	g	22	14
Added sugar	g	50	33
Sodium	mg	2,000	1,300
**Nutrients to encourage**			
Protein	g	50	32.5
Dietary fiber	g	25	15
**Vitamins**		**NRV**	**NRV**
Vitamin A	μg	800	800
Vitamin D	μg	5	5
Vitamin E	mg	12	12
Vitamin K	μg	75	75
Vitamin C	mg	80	80
Thiamin	mg	1.1	1.1
Riboflavin	mg	1.4	1.4
Niacin	mg	16	16
Vitamin B6	mg	1.4	1.4
Folate	μg	200	200
Vitamin B12	μg	2.5	2.5
Pantothenate	mg	6	6
Biotin	μg	50	50
**Minerals**			
Calcium	mg	800	800
Phosphorus	mg	700	700
Magnesium	mg	375	375
Iron	mg	14	14
Zinc	mg	10	10
Iodine	μg	150	150
Copper	mg	1	1
Selenium	μg	55	55
Manganese	mg	2	2
Molybdenum	μg	50	50

### 2.6 Selection of FNC nutrients and ingredients to encourage and nutrients to limit

Protein and fiber are nutrients to encourage, consistent with Nutri-Score, the HSR and the NRF family of scores (42, 48, 49). Desirable ingredients, as featured in many nutritional recommendations (56, 57) and found in snack products, include dairy components, nuts and seeds, fruit, whole grains. Those ingredients contain vitamins and minerals, as well as poly-unsaturated fatty acids in nuts, bioactive polyphenols and flavonoids in chocolate and tea. Vegetables and legumes are less common ingredients, though there are emerging categories of vegetable chips and pulse-based snacks (58, 59).

Consistent with most dietary guidelines, nutrients to limit are saturated fat, added sugar and sodium. For clarity, added sugars are defined as sugars added to food during food processing, sugars used as sweeteners and sugars from honey and concentrated fruit or vegetable juices. Added sugars do not include naturally occurring sugars, such as sugars in the intact cell walls of fruit and vegetables, or sugars present in milk (60). For snacks other than fruit, milk and derived products, the content of total and added sugars is quite similar.

Industrial trans-fats (iTFAs) are another nutrient to limit. Since 2006, Ferrero stopped using partially hydrogenated fats in Ferrero brand and Kinder products. In May 2019, Ferrero signed the International Food and Beverage Alliance (IFBA) Global iTFAs Commitment to limit the amount of iTFAs in all products (51). This guideline has been implemented to ensure that the amount of iTFAs in all products does not exceed 2 g per 100 g of fat/oil (51). For the ambient bakery and sweet biscuits sub-categories, Ferrero signed the Global Sodium Reduction Commitment in 2021 (51). This agreement established voluntary minimum sodium reduction targets for members of the IFBA.

### 2.7 The importance of progressive FNC values

A continuous set of nutrition criteria, as opposed to a pass or fail score, allows for better monitoring of progress toward nutrition goals. There are advantages to assessing step-by-step progressive improvements in product formulation, making it easier to improve the nutritional features of the products. By focusing on small achievable changes within a given product category, a progressive set of criteria is better suited to the continuous innovation and formulation of product lines. This method can also help set incremental goals for nutrients to limit and for the nutrients and ingredients to encourage. These concepts are summarized in Table 2.

**TABLE 2:** A summary of considerations taken in developing the Ferrero Nutrition Criteria (FNC).

Category specific	Base of calculation: caloric target	Nutrients and ingredients to encourage	Nutrients to limit	Progressive step-by-step approach
– Tailored nutritional criteria: consider the different types of foods, their role in the diet, and how they are consumed. – Takes into account the specific nutritional context of each food category leading to realistic formulation targets. – Helps identify items that are best within a given category. – Encourages the development of new products that align with dietary guidelines and consumer expectations. – Ensures that products are formulated to fit within reasonable calorie limits, promoting responsible consumption patterns.	– Products are individually packaged for consumption with or between meals. – Reflects realistic consumption patterns and more accurate calorie intake per eating occasion. – Encourages product formulation to create products that fit within a reasonable calorie limit, promoting responsible consumption. – Formulating per caloric target provides flexibility and ensures challenging yet achievable progress.	– Consistent with most dietary guidelines, it is important to encourage nutrients like protein, dietary fiber, vitamins, and minerals, bioactive compounds, as well as ingredients such as dairy, nuts, fruits, whole grain, legumes, seeds and vegetables.	– Typically include energy, saturated fat, trans-fat, total or added sugar, and sodium which are linked to non-communicable diseases when consumed in excess.	– Allows for step-by-step improvements in product formulation, making it easier to improve nutritional features of products. – Provides flexibility by setting incremental goals for nutrients to limit and encourage. – By focusing on small, achievable changes, promotes continuous innovation of products to improve their nutritional quality.

## 3 Results

As described below, the FNC categorizes products into 8 different categories with 13 sub-categories based on the type of product, nutrient and ingredient composition, consumption occasion, and the product’s role in the total diet.

### 3.1 Product categories and serving sizes

The FNDDS food categories covered many Ferrero product lines. Analyses of FNDDS 2017-18 data first established median FDA serving sizes (i.e., RACC values). There were also calculated median energy, added sugar, saturated fat, and protein content by food category, all in grams per serving. Table 3 shows FNDDS and Ferrero food categories, median RACC values and median calories per RACC in the FNDDS database. Ferrero average serving sizes are indicated as well.

**TABLE 3:** Ferrero product categories and FNDDS food groups and subgroups.

Ferrero categories	Ferrero sub-categories	Ferrero servings (mean g)	Target kcal/serving	FNDDS 2017-18 categories	Mean RACC	Median RACC	Median kcal/serving
Edible Ices	Ice cream milk-based	70	≤ 250 kcal	Ice cream; frozen desserts	100	100	215
	Water ices and sorbets	80	≤ 100 kcal	Gelatins, ices, sorbets	104	120	67
Fine Bakery Wares	Ambient bakery	30	≤ 200 kcal	Doughnuts, sweet rolls, pastries	74	55	228
				Cakes and pies	90	80	306
	Sweet biscuits	30	≤ 200 kcal	Cookies and brownies	30	30	135
Chilled	Chilled snacks	30	≤ 200 kcal	Pudding	120	120	169
Beverages	Ready-to-drink tea	200	≤ 70 kcal	Non-dairy drinks	240	240	120
Sugar confectionery	Hard candy	2	≤ 10 kcal	Candy without chocolate	25	30	118
	Soft candy	10	≤ 50 kcal	Candy without chocolate	25	30	118
Confectionery	Spreads	15	≤ 100 kcal	Nut spreads	37	37	221
	Chocolate confectionery	30	≤ 200 kcal	Candy with chocolate	28	30	147
	Pralines and Bites	10	≤ 100 kcal	Candy with chocolate	28	30	147
Protein bar	Low-sugar protein bar	55	≤ 250 kcal	Nutrition bars	40	40	165
Fruit and nuts snacks	Fruit and nuts bar and trail mix	40	≤ 250 kcal	Dried fruits	40	40	118

Median RACC (g) and median energy per serving (kcal/RACC) are shown by category. Ferrero average serving sizes are indicated as well.

In general, Ferrero average serving sizes were smaller than the government mandated FDA RACC values in the US. Ferrero average serving size for ready-to-drink tea was 200 g as opposed to 240 g in the US. Ferrero average values for edible ices, ambient bakery, chilled snacks, sugar confectionery, pralines and bites were lower than the RACC values in the US. The US RACC for nut spreads is 2 tbs or 37 g, whereas Ferrero’s suggested serving is 15 g. These differences in serving size have implications for the calculations of energy and nutrients per serving. Serving sizes were the same or comparable to the RACC for several food sub-categories, notably sweet biscuits, chocolate confectionery, protein bars, fruit and nuts bar and trail mix.

Based on the above analyses and considering that these food categories should account for up to 10% of the daily caloric intake within the overall diet for both adults and children (58, 59, 61–64), Ferrero set a limit for the energy content of fine bakery wares, chilled snacks, and confectionery to under 200 kcal/serving for adults and older children and 130 kcal/serving for children aged 3–8 years. For ice cream, protein bars and fruit and nuts snack the limit was set to under 250 kcal/serving for adults and older children and 150 kcal/serving for children aged 3–8 years. These aims are consistent with the data presented below. Most snack categories were below 200 kcal/serving, other than cakes and pies, doughnuts, sweet rolls and pastries. Chocolate candy and sugar candy in particular provided less than 150 kcal/serving on average.

### 3.2 Nutrient content per 100 g by category in FNDDS 2017-18

**FIGURE 1:**
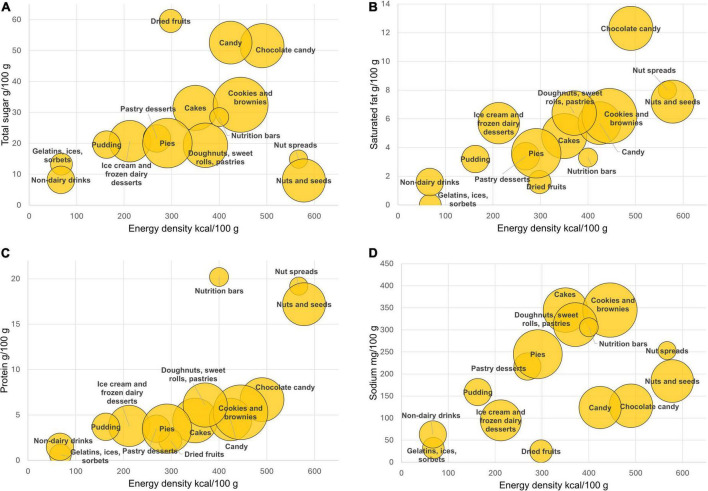
FNDDS mean values for total sugar **(A)**, saturated fat **(B)**, protein **(C)**, and sodium **(D)** expressed per 100 g and plotted against energy density (kcal/100 g) by FNDDS food category. The size of the bubble corresponds to the number of items in each food category.

Mean energy and selected nutrient content by product category are shown in Figure 1. First, energy density values (kcal/100 g) covered a wide range from gelatins and sorbets to nuts and seeds. The energy density of chocolate was in the order of 500 kcal/100 g, while the energy density of cakes and pies was closer to 300 kcal/100 g.

Total sugar content per 100 g was highest for dried fruit followed by candy and chocolate candy. The saturated fat content was highest for chocolate candy followed by nuts and seeds and nut spreads. The protein content was highest for nutrition bars, followed by nut spreads and by nuts and seeds. Sodium content was highest for bakery goods: cookies and brownies, cakes and doughnuts, sweet rolls, pies and pastries. Nutrition bars were relatively high in sodium.

### 3.3 Nutrient content per serving by category in FNDDS 2017-18

**FIGURE 2:**
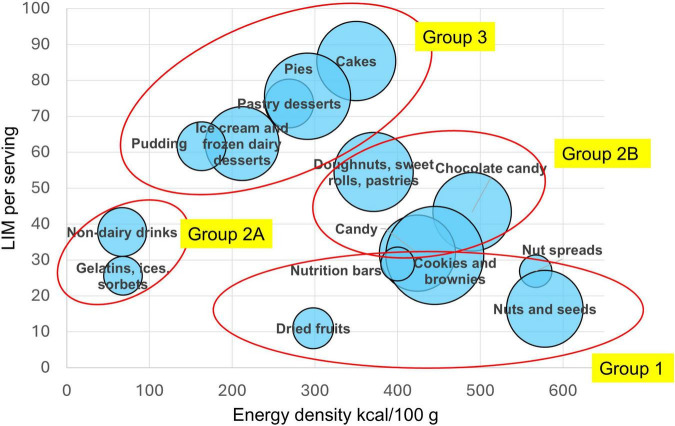
Mean nutrients to limit (LIM) score values plotted against energy density (kcal/100 g) by food category. The size of the bubble corresponds to the number of items in each food category.

Many products in the snacks category are not only energy dense but can also be high in saturated fat, sugar and sodium. Most nutrient profile models use a negative sub-score composed of the three nutrients to limit (LIM). This composite sub-score has been calculated per 100 g, per 100 kcal, or per serving (65). The present calculations show the LIM sub-score expressed per serving or RACC. Figure 2 shows composite LIM sub-score values per serving plotted against energy density by food category. The lowest median LIM scores were obtained for dried fruits, nuts and seeds, nutrition bars, nut spreads, gelatins, and sorbets. Higher LIM values were obtained for non-dairy beverages, cookies and brownies. Doughnuts, sweet rolls and pastries had even higher LIM scores. The highest LIM values were obtained for cakes, pies, pastry desserts, puddings and ice creams. These products tended to be high in both sugar and saturated fat.

As shown in Figure 2, the products can be separated into distinct groups based on their energy density and the per serving content of saturated fat, total sugar and sodium. This visualization was the basis of the proposed classification of foods and beverages in the global Ferrero product portfolio.

## 4 Proposed classification of Ferrero products

**FIGURE 3:**
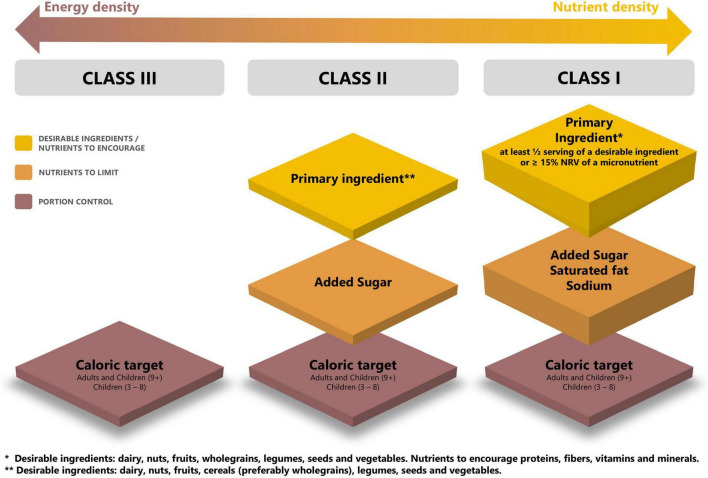
Architecture of Ferrero Nutrition Criteria (FNC).

FNC was used to assign products into three classes (Figure 3), each with specific guidelines and criteria. Nutrient density improves and energy density is reduced with stepwise progression from Class III to Class I. The current goal is to ensure that Ferrero products within each category remain best of class in terms of taste but also nutritional value. Each class is defined with criteria based on maximum levels for portion control and nutrients to limit, and minimum levels for desirable ingredients and nutrients to encourage. Products in each class are marketed in small portion sizes, providing about 200 kcal per serving or less, with some exceptions made for ice cream milk-based, protein bars, fruit and nuts snacks due to their specific composition and frequency of consumption. The category specific approach facilitates product formulation and innovation.

### 4.1 Ferrero Nutrition Criteria—class III

Class III foods are generally energy-dense and are formulated with a primary focus on taste, which is the main driver of consumer expectations and consumer food choice (2). Given the principle that “all foods can fit,” these items can be part of a balanced diet if consumed in moderation and as part of an active lifestyle. To promote responsible consumption, these foods are offered in small portion sizes. Additionally, given the recognition that a single snack should provide no more than 10% of daily calories to a healthy diet (58, 59, 61–64), a caloric target of approximately 10% DV for energy per serving was set, depending on food category (Table 4).

**TABLE 4:** Ferrero Nutrition Criteria (FNC): pre-requisites, caloric target, nutrients to limit, nutrients and ingredients to encourage for product categories and subcategories referred to adults and children 9 + years and children aged 3–8 years according to the class.

Category	Subcategory	Target	CLASS	Pre-requisites		Caloric target (kcal/serving)	Nutrients to limit			Nutrients and ingredients to encourage	
				iTFA (g/100 g fat/oil)	Sodium (mg/100 g)		Added sugar (%DV/serving)	Saturated fats (%DV/serving)	Sodium (%DV/serving)	Fiber (g/100 g)	Desirable ingredients
Edible ices	Ice cream milk-based	Adults and children (9 +)	Class I	≤ 2		≤ 120	≤ 15	≤ 14	≤ 6	–	*
			Class II			≤ 200	≤ 20	–	–	–	**
			Class III			≤ 250	–	–	–	–	–
		Children (3–8)	Class I			≤ 100	≤ 15	≤ 14	≤ 6	–	*
			Class II			≤ 130	≤ 20	–	–	–	**
			Class III			≤ 150	–	–	–	–	–
	Water ices and sorbets	Both	Class I			≤ 60	≤ 15	≤ 2	≤ 6	–	*
			Class II			≤ 100	≤ 20	–	–	–	**
			Class III			≤ 100	–	–	–	–	–
Fine bakery wares	Ambient bakery	Adults and children (9 +)	Class I	≤ 2	≤ 300	≤ 150	≤ 15	≤ 10	≤ 6	≥ 3	*
			Class II			≤ 200	≤ 20	–	–	–	**
			Class III			≤ 200	–	–	–	–	–
		Children (3–8)	Class I			≤ 130	≤ 15	≤ 10	≤ 6	≥ 3	*
			Class II			≤ 130	≤ 20	–	–	–	**
			Class III			≤ 130	–	–	–	–	–
	Sweet biscuits	Adults and children (9 +)	Class I		≤ 380	≤ 150	≤ 15	≤ 10	≤ 6	≥ 3	*
			Class II			≤ 200	≤ 20	–	–	–	**
			Class III			≤ 200	–	–	–	–	–
		Children (3–8)	Class I			≤ 130	≤ 15	≤ 10	≤ 6	≥ 3	*
			Class II			≤ 130	≤ 20	–	–	–	**
			Class III			≤ 130	–	–	–	–	–
Chilled	Chilled snacks	Adults and children (9 +)	Class I	≤ 2		≤ 150	≤ 15	≤ 14	≤ 6	–	*
			Class II			≤ 200	≤ 20	–	–	–	**
			Class III			≤ 200	–	–	–	–	–
		Children (3–8)	Class I			≤ 100	≤ 15	≤ 14	≤ 6	–	*
			Class II			≤ 130	≤ 20	–	–	–	**
			Class III			≤ 130	–	–	–	–	–
Beverages	Ready-to-drink tea	Both	Class I	≤ 2		–	–	–	–	–	*
			Class II			≤ 70	Sugar free	–	–	–	**
			Class III			≤ 70	–	–	–	–	–
Sugar confectionery	Hard candy	Adults and children (9 +)	Class I	≤ 2		–	–	–		–	*
			Class II			≤ 10	Sugar free	–	–	–	**
			Class III			≤ 10	–	–	–	–	–
	Soft candy	Adults and children (9 +)	Class I			–	–	–		–	*
			Class II			≤ 50	Sugar free			–	**
			Class III			≤ 50	–	–	–	–	–
Confectionery	Spreads	Both	Class I	≤ 2		–	–	–	–	–	*
			Class II			≤ 100	≤ 11	–	–	–	**
			Class III			≤ 100	–	–	–	–	–
	Chocolate confectionery	Adults and children (9 +)	Class I			–	–	–	–	–	*
			Class II			≤ 200	≤ 20	–	–	–	**
			Class III			≤ 200	–	–	–	–	–
		Children (3–8)	Class I			–	–	–	–	–	*
			Class II			≤ 130	≤ 20			–	**
			Class III			≤ 130	–	–	–	–	–
	Praline and bites	Both	Class I			–	–	–	–	–	*
			Class II			≤ 100	≤ 10			–	**
			Class III			≤ 100	–	–	–	–	–
Protein bar	Low-sugar protein bar	Adults and children (9 +)	Class I	≤ 2		≤ 200	Total sugar ≤ 5 g/100 g	≤ 14	≤ 6	–	*
			Class II			≤ 250	Total sugar ≤ 5 g/100 g	–	–	–	**
			Class III			–	–	–	–	–	–
Fruits/nuts snacks	Fruit/nuts bars and trail mix	Adults and children (9 +)	Class I	≤ 2		≤ 200	≤ 10	≤ 14	≤ 6	≥ 3	*
			Class II			≤ 250	≤ 25	–	–	–	**
			Class III			–	–	–	–	–	–
		Children (3–8)	Class I			≤ 100	≤ 10	≤ 11	≤6	≥ 3	*
			Class II			≤ 130	≤ 20	–	–	–	**
			Class III			–	–	–	–	–	–

*Class I - The first ingredient must be one of the following: Dairy (D), Nuts (N), Fruits (F), Whole Grains (WG), Legumes (L), Seeds (S), or Vegetables (V). It should also meet one of the following criteria: - It must contain at least 1/2 serving of D/N/F/WG/L/S/V. For mixed products, each food group added should be at least equal to 1/4 serving. - It should provide at least 15% of the Nutrient Reference Value (NRV) of a micronutrient of concern per 100 g. Foods that have D/N/F/WG/L/S/V as the first ingredient after water also qualify. In protein bar category, protein is allowed as the first ingredient. **Class II—The first ingredient must be one of the following: D/N/F/L/S/V/C preferably WG. Cereals (C). In protein bar category, protein is allowed as the first ingredient.

Protein bars and fruit and nut snacks were excluded from Class III because of their nutritional composition and role in the diet. However, protein bars and fruit and nut bars can serve as a model of how the nutritional value of Class III products can be improved by the addition of desirable ingredients. Notably, certain Class III foods could provide nutrients to encourage due to the presence of desirable ingredients such as proteins from milk and dairy products, fiber and fats from nuts. However, these foods fall into Class III because they do not meet the stricter criteria of the other classes detailed below.

### 4.2 Ferrero Nutrition Criteria—class II

Class II foods are formulated to strike a balance between taste and nutritional value. To promote responsible consumption, these products are consumed in small portion sizes, with a caloric target set at around 10% DV for energy per serving, depending on food category. To improve the nutritional value of this class compared to Class III, targets were set for limiting certain nutrients and incorporating desirable ingredients. Specifically, products need to contain less than or equal to 20–25% DV of added sugars per serving, depending on product category. For low-sugar protein bars, the limit for total sugar is set in accordance with European regulations, which mandate that the claim low sugars may only be made if the product contains no more than 5 g of sugars per 100 g (66). Additionally, the first ingredient should be one from desirable food groups to encourage according with Food-Based Dietary Guidelines (57), including dairy, nuts, fruits, cereals preferably whole grains, legumes, seeds, and vegetables (Table 4). These additional parameters were chosen to ensure that Class II foods not only meet taste expectations but also contribute positively to a balanced diet by providing essential nutrients and limiting less desirable ones.

### 4.3 Ferrero Nutrition Criteria—class I

Class I products embody the concept of nutrient density, as supported by WHO and FDA (1, 35), emphasizing the importance of foods that offer more nutrients relative to calories, without compromising taste. To promote responsible consumption, these products are consumed in small portion sizes with a caloric target set at less than or equal to 10% DV for energy. Compared to Class II, the limits for added sugars have become more stringent, and criteria for saturated fats and sodium have been added. Specifically, added sugars and saturated fats should be less than or equal to 15% DV, and sodium should be less than or equal to 6% DV per serving, depending on the product category. For low-sugar protein bars, the limit for total sugar is set at no more than 5 g per 100 g, in compliance with European regulations (66). Class I products should adhere to various criteria that promote nutritional improvement by encouraging desirable nutrients and ingredients, depending on the specific category. For some categories, criteria for desirable nutrients have been established, for example, protein bars should provide a relevant amount of protein according to specific local regulations, while fine bakery wares and fruit and nut snacks should contain at least 3 grams of fiber per 100 grams. To meet the criteria for Class I, products must contain as their first ingredient one from desirable food groups, including milk and dairy, nuts, fruits, whole grains, legumes, seeds and vegetables, in accordance with most Food-Based Dietary Guidelines (57). Table 5 illustrates the reference amounts for these desirable ingredients. The minimum inclusion criterion for enhancing nutrient density is set at 1/2 serving, with the option to combine smaller portions from multiple food groups to achieve this threshold (e.g., 1/4 serving of dairy plus 1/4 serving of nuts). Alternatively, products should provide a significant amount of at least one micronutrient of concern, defined as 15% NRV per 100 g (Table 4). These additional parameters ensure that the products not only meet taste expectations but also improve their nutritional value. Beverages, sugar confectionery, and confectionery categories are excluded from Class I because their inherent nature does not align with the characteristics of this class, making them more suitable for other classes that place a greater emphasis on taste.

**TABLE 5:** Desirable ingredients to encourage and their specific requirements.

Desirable ingredients	1 Serving	1/2 Serving	1/4 Serving
Dairy	*Milk: 200 mL	100 mL	50 mL
	Yoghurt: 125 g	62.5 g	31 g
Nuts	Nuts: 30 g	15 g	7.5 g
Fruits	Fresh fruit: 80 g	40 g	20 g
	Dried fruit: 30 g	15 g	7.5 g
	Fruit juice: 150 mL	75 mL	37.5 mL
Whole grains	Whole grains: 16 g	8 g	4 g
Legumes	Dried legumes: 30 g	15 g	7.5 g
Seeds	Seeds: 30 g	15 g	7.5 g
Vegetables	Vegetable: 80 g	40 g	20 g

*For skimmed and whole milk powder, the rehydration factor for reconstitution is taken into consideration.

## 5 Discussion

The food industry as a whole has been developing nutrition standards to create healthier product portfolios. Such standards need to be transparent, published, and open to public scrutiny. Communicating both current and proposed nutrient standards developed by Ferrero was the purpose of this report.

The Ferrero global product portfolio encompasses several distinct product categories. These are edible ices, fine bakery wares, chilled products, beverages, sugar confectionery, confectionery, protein bar, fruit and nut snacks (67). The Ferrero products include packaged snacks but can also be consumed as components of structured eating occasions. These categories are broadly similar but not identical to the food categories selected for analysis from the USDA FNDDS nutrient composition database. Sometimes databases lag behind new product acquisition or development. In recent years, Ferrero has made acquisitions in the area of protein bars, and fruit and nut bars (67). Ferrero products are available in 170 countries worldwide, including those in the European Union and North America.

The present analyses aligned Ferrero product lines with the food groups, subgroups and categories found in the USDA FNDDS 2017-18 nutrient composition database. The FNDDS food categories did include chocolate, sugar confectionery, cookies, other bakery goods, ice creams and sorbets, nut spreads, dairy desserts and non-dairy beverages (35). Among food categories examined were also dried fruit and nuts and seeds. The food categories were analyzed for energy and nutrient content per serving and were profiled using a version of the NRF score, using servings as the reference amount. Indeed, many industries driven nutrient profiling systems are calculated per serving rather than per 100 g as the reference amount. For example, some NPMs use serving sizes for dishes, snacks or condiments that are characteristics of their region (19, 68). Calculations by serving size are especially important for snacks that are not part of the core diet but are meant to be eaten infrequently and in small portions.

Based on these analyses and considering the role of these food categories in the diet, Ferrero has developed a set of own category-specific nutrition criteria. The FNC was developed primarily for internal use to help evaluate the quality of the existing portfolio and to help guide the development and formulation of new products. The present guidance is especially relevant to packaged snacks that are often individually wrapped in single serving sizes. The FNC will be the tool used to set realistic, impactful nutrition targets, guide innovation and explore potential market niches. As documented in this report, product lines were assigned into 3 classes, based on the type of product, their energy and macronutrient content. Individual categories of food and beverage products were assigned criteria for calories, saturated fats, iTFA, added sugar and sodium. Likewise, positive nutrients were assigned with the overall intent to improve nutritional value among classes and products.

The present FNC contributes to the growing literature on the uses of nutrient profiling by the food industry. The initial purpose of nutrient profiling, as described in early literature, was to help consumers make healthier food choices at the point of sale (13, 69). Early NPMs (46, 49, 70) were consumer-facing and were adopted by supermarket chains (71). Such models had value in assisting government policies aimed at health promotion and disease prevention (72). However, it soon became clear that nutrient profiling of product lines could also be used by the food industry to guide innovation and reformulation of foods and beverages (73–75). Industry driven models have focused on positive nutrition, featuring protein, fiber, vitamins and minerals, and desirable ingredients, while also addressing nutrients of public health concern, such as excessive saturated fat, added sugar and sodium. Those NPMs, created for internal use (76) were consistent with WHO guidance on improving the quality of the food supply. At this point, nutrient profiles continue to provide the scientific basis for nutrient content claims, front-of-pack labels, and regulating advertising and marketing to children (76). But perhaps the main value of nutrient profiling is to help guide the formulation of food products and new product development.

For example, the progressive Greenberg et al. model was designed for internal use only (15), to guide and monitor continuing improvements in the overall nutritional quality of foods and beverages. The criteria were based on likely frequency of consumption, serving sizes, and the place of the food or beverage in the total diet (15). This model assigned food products into four classes of increasing nutritional value, based on the content of nutrients to limit, along with nutrients and ingredients to encourage (15). Category-specific progressive standards were proposed for calories, sodium, added sugars, saturated fats, and for iTFAs. This model also listed minimum values for low-fat dairy and for desirable MyPlate food ingredients for potential inclusion in new products.

Global nutrition targets developed in another study were also intended for internal use, with a focus on formulation of condiments and savory snacks that are consumed infrequently and in small portions (18). The category-specific nutrition targets were intended to guide the development of healthier product lines that were lower in saturated fats, total sugars, and sodium, and contained desirable ingredients. The targets took into account product use patterns as well as regulatory, technological, sensory and safety constraints (18).

What the various industry driven systems have in common is that they are category-specific, with nutrient standards tailored to different food groups. Across-the-board NPMs are not as effective when it comes to product reformulation, as they fail to account for the unique nutritional compositions and requirements of different categories. Category-specific models are more helpful when it comes to new product development, especially when they overlap with product lines. Models that capture incremental improvements are more useful than pass or fail scores that do not indicate what could be done better.

The FNC has limitations. Assessing the potential impact on public health is complex due to the interplay of various factors and independent variables. Consequently, the public health implications of the FNC were beyond the scope of this paper. The USDA databases are extensive, detailed, and publicly available. The database used in the present analysis was aligned with food consumed in the nationally representative US NHANES, enabling future analysis of the link between foods, diets, and health. However, this approach has limitations, as it does not account for the diverse dietary patterns of different populations. Additionally, the current FNC considers only two consumption targets, without addressing the specific nutritional needs of other target groups. The FNC is not intended to be consumer-facing and is not designed to educate consumers on the nutritional quality of products. Such a system will need to be updated regularly to respond to changes in dietary guidance. Continuous evaluation and adaptation of the model will be crucial in meeting evolving nutritional standards and consumer expectations.

## 6 Conclusion

The newly developed FNC assigns sweet-packaged foods and beverages into three classes, based on energy and nutrients to limit per serving and on nutrients and desirable ingredients to encourage. The FNC, intended primarily for internal use, will help with assessing the product portfolio, guide innovation, new product development and formulation. The FNC is not static, it is intended to evolve continually, incorporating feedback from stakeholders and consumers and adapting possibly to the evolution of dietary guidelines and health recommendations. For instance, we acknowledge that environmental sustainability is an increasingly important consideration in food system transformation. Integrating such criteria remains an important avenue for future research.

## Author contributions 

FC: Formal Analysis, Writing – review and editing, Methodology, Data curation, Project administration, Writing – original draft, Investigation, Visualization. MC: Writing – review and editing, Supervision. AD: Writing – original draft, Formal Analysis, Methodology, Data curation, Visualization, Investigation, Writing – review and editing, Validation. EA: Investigation, Writing – review and editing, Data curation. MB: Writing – review and editing. EC: Writing – review and editing, Supervision. MF: Writing – review and editing, Supervision. SG: Writing – review and editing. FM: Investigation, Data curation, Writing – review and editing. MM: Writing – review and editing. IM: Visualization, Writing – review and editing. GR: Writing – review and editing. VR: Data curation, Investigation, Writing – review and editing. GV: Writing – review and editing, Investigation, Data curation. SC: Writing – original draft, Project administration, Conceptualization, Supervision, Writing – review and editing. EP: Conceptualization, Writing – review and editing.

## Data Availability

The original contributions presented in the study are included in the article/supplementary material, further inquiries can be directed to the corresponding authors.
